# Effect of Lightweight Aggregate Impregnation on Selected Concrete Properties

**DOI:** 10.3390/ma15010198

**Published:** 2021-12-28

**Authors:** Lucyna Domagała, Agnieszka Podolska

**Affiliations:** Faculty of Civil Engineering, Cracow University of Technology, 31-155 Cracow, Poland; aga.podolska@gmail.com

**Keywords:** lightweight concrete, lightweight aggregate, aggregate impregnation, sintered fly ash, expanded clay, moisture content, concrete drying, compressive strength, water absorption

## Abstract

The impregnation of lightweight aggregate (LWA) is an alternative method to its pre-moistening, which is used to limit the loss of fresh concrete workability due to the aggregate’s ability to absorb a great amount of mixing water. The aim of this study was to access the effectiveness, by pre-coating LWAs with cement paste, in modifying the properties of concrete composites. Two types of lightweight aggregates (Lytag and Leca) characterized with a relatively open-structure shell were selected. The other changeable parameters taken into consideration in this research were: LWA size, initial moisture of aggregate before the impregnation process and type of cement paste applied as an impregnant. Sixteen concretes prepared with pre-moistened and pre-coated lightweight aggregates were subject to a density test in different moisture conditions, a water absorption test and a compressive strength test. On the one hand, the pre-coating of LWAs with cement paste resulted in a relatively slight increase in concrete density (by up to 19%) compared to the pre-moistening of LWAs. On the other hand, it caused a very significant reduction (by up to 52%) in the composite’s water absorption and an incomparably greater growth (by up to 107%) in compressive strength. The most crucial factors determining the effectiveness of impregnation of LWAs with cement pastes in improvement of composite properties were the aggregate type and its size. The composition of impregnating slurry and the initial moisture content of LWA before pre-coating also mattered.

## 1. Introduction

Lightweight aggregate concrete (LWAC) is one of the most versatile building materials. It is used for both structural and insulating purposes. Due to some technological problems resulting from the porous nature of lightweight aggregate (LWA), precast applications of the material are preferable and much more common in practice in comparison to the monolithic ones [1,2,3]. The most important difficulties connected with LWA application are high risk of aggregate segregation, workability loss and problems with pumping transportation [1,4]. All the above-mentioned problems may be eliminated or minimalized by special procedures of fresh concrete preparation, placing and compaction, which are easier to be executed in conditions of precast plants. Some of the basic and commonly used LWA treatments are initial aggregate wetting, resulting in limitation or total elimination of aggregate absorption of mixing water from fresh concrete, and increasing the content of water in concrete by the amount resulting from aggregate water absorption. Meanwhile, there is a lot of evidence [5,6,7,8,9] showing deterioration of LWAC performance when it is prepared with initially moistened or saturated aggregate or with higher content of water. In particular, lightweight concretes prepared with pre-saturated aggregate characterized by higher water absorption (WA_24h_ ≥ 10–15%) may reveal worse durability: higher chloride penetration and carbonation, lower freeze-thaw resistance and water tightness [5,6,7,8,9].

The other method to limit the negative consequences of high water absorption of lightweight aggregate in fresh concrete is LWA impregnation. The technology of initial pre-coating of porous aggregate with certain compounds has been well-known for decades. Nevertheless, it is rarely used due to its higher costs in comparison to LWA pre-saturation. Meanwhile, such an initial aggregate preparation does not cause worse concrete performance, as pre-saturation may. Additionally, in contrast to non-impregnated LWA, the pre-coating of lightweight aggregates makes it possible to determinate the exact value of the effective water-cement ratio (*w*/*c*) and to estimate LWAC properties on the basis of its composition. As was proved in [10], the estimation of effective water-cement ratio according to EN 206 [11] does not give sufficient accuracy in the case of lightweight concrete prepared with LWA of higher water absorption and/or moistened to a relatively low initial moisture content in relation to its water absorption. 

On the one hand, a lot of research [12,13,14,15,16,17,18,19,20] proved that application of initially impregnated LWAs may result in lower cement content, stabilization of concrete workability, the decrease in concrete water absorption, shorter time of element/structure drying out and the increase in concrete strength and durability as well as thermal and acoustic insulation. On the other hand, LWAs impregnation may restrain internal curing with water accommodated in aggregate, the increase in concrete autogenous shrinkage, the increase in a rate of drying shrinkage and the increase in density, as well as deterioration of the bond between aggregate and cement paste [12,13,15]. It should be noted that qualitative and quantitative effect of aggregate sealing is dependent on both properties of aggregate and impregnation procedures. 

There are many methods applied for impregnation of porous aggregates. They are usually immersed in any liquid medium, or some compounds are sprayed on their surfaces. The above-mentioned processes are performed once or repeated many times. To improve the aggregates tightness, porous aggregates may be impregnated with different substances, e.g., natural or synthetic polymers [12,13,16,17,18,19,21,22], kerosene, different oils [13], paraffin [12,14,23,24,25,26], sodium carbonate solutions [27,28] or sodium silicate [13,18,29], calcium metasilicate [15] and cement paste [13,20,21,29,30,31,32]. Besides a sealing effect, in some cases the main aim of submergence of porous aggregates in paraffin or polyethylene glycol is to deliver a phase change material to concrete composite in order to improve its freeze-thaw resistance [26] or thermal properties [12,14,24]. On the other hand, pre-coating LWAs with silicates or carbonates solutions are used not only due to the enhancement of LWAs tightness but also the self-healing effect [18,27,28]. It should be stated that in opposition to recycled aggregate or natural organic one, the impregnation process used for natural or manufactured LWAs of mineral origin does not require additional treatments, e.g., soaking in acid, in silica fume or nanosilica solution, calcic lime solution or cement paste to enhance the bond between aggregate surface and matrix in a composite [15,21,23,33]. 

Cement paste used for aggregate impregnation may be a plain cement grout or a mix of cementitious materials (i.e., cement, fly ash, silica fume, ground granulated blast furnace slag, ground colemanite) and admixtures (typically plasticizers or superplasticizers) [13,20,21,29,30,31,34,35]. Nevertheless, there are only a few examples of research dedicated to the impregnation of LWA with cement paste. Mostly, these studies are focused on natural mineral or organic lightweight aggregates, e.g., [12,19,31]. Generally, pre-coating with cement paste is more often considered in the case of recycled aggregates, e.g., [21,29,30,31]. In the case of lightweight aggregates, polymers are deemed to be more effective for sealing than cement pastes. Moreover, there are some doubts that the procedure of pre-coating LWAs with cement paste may take a longer time or cause s higher increase in density in comparison to impregnation with polymers. Whereas, pre-coating LWAs with cement paste may result in a better bond between aggregate and cement matrix in composite due to excellent material compatibility [32]. Additionally, filling external pores with cement paste, which is usually a denser and stronger material than the other impregnants, may lead to lower risk of fresh concrete segregation and higher hardened composite strength. Few studies [13,20] comparing the effectiveness of various impregnating substances used for pre-coating porous aggregates showed that, in comparison to sodium silicate solution, white latex solution and wood oil, cement paste may be more effective in reduction of LWAC water absorption. Although the impregnation with waterproof agent solution caused a decrease in water absorption of concrete, the effect of this impregnant on compressive strength and modulus of elasticity enhancement turned out to be comparable to or worse than pre-coating with cement paste. However, the assessment of durability of LWAC made of aggregate impregnated with different substances, subject to magnesium sulfate solution exposure, showed that cement paste pre-coating protected concrete most efficiently. 

It was proved in [32] that the main parameter influencing the effectiveness of impregnation of LWAs with cement paste on the aggregate properties is LWA type. That is why the impregnation of lightweight aggregates with a strongly sintered external shell, e.g., Liapor, did not lead to any pronounced effects, while for aggregates with a more open structure of external shell, e.g., tested Lytag and Leca, the treatment with cement paste resulted in significant enhancement of their initial tightness. The water absorption of these modified aggregates were reduced by ca. 2–4 times in relation to plain aggregates, while crushing resistance was increased by only up to over a dozen percent. Other factors of impregnation, such as LWA particle size and its initial moisture content, as well as type of cement paste used for pre-coating, turned out to be of secondary importance for the properties of modified aggregates. Nevertheless, it should be stated that assessment of impregnation effectiveness on aggregate properties and composite properties may vary greatly in terms of quantity. Tests of concretes prepared with recycled aggregate pre-coated with pozzolanic powder revealed that the relatively lower water to binder ratio of cement paste used for coating the aggregate benefits greatly from a denser interfacial transition zone and, hence, a higher strength [34]. Therefore, it should be expected that the impact of the type of cement paste used for LWAs impregnation should also have an impact on lightweight concrete properties. 

The aim of this research was to access the influence of pre-coating lightweight aggregate with cement pastes on selected properties of concrete. In relation to the studies referred to in this Introduction, the effect of aggregate impregnation was researched taking into consideration both properties of LWAs and properties of cement pastes. Additionally, lightweight aggregates were subject to pre-coating in various initial moisture conditions.

## 2. Materials and Methods

**Figure 1:**
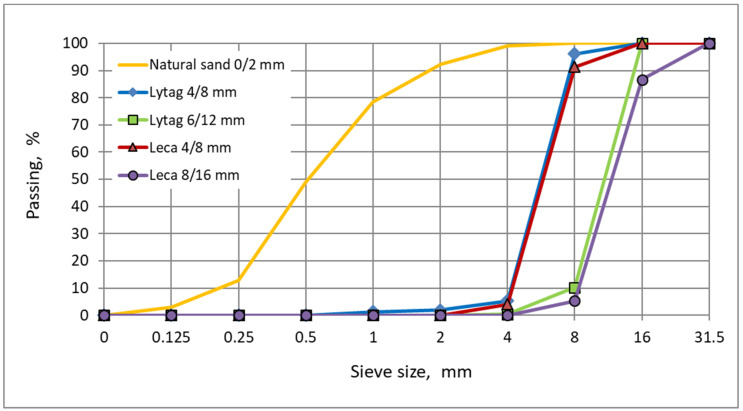
Grading curves for natural sand and lightweight aggregates.

The research program covered 16 concretes made of lightweight aggregates, pre-coated with cement pastes and plain LWAs as reference. Two different types of lightweight aggregates characterized with an open-structure shell were selected for tests. The first type was sintered fly ash Lytag, with particle density of 1320–1340 kg/m^3^ and maximum water absorption of 24–25%. The second type, expanded clay Leca, was much more porous, therefore, its particle density was only 550–560 kg/m^3^, while water absorption was as high as 32–41%. Two coarse fractions were chosen from each LWA type to prepare concretes: 4/8 mm and 6/12 mm or 8/16 mm. The properties of all 4 plain lightweight fractions and 12 impregnated aggregates used for the research are given in Table 1 and Figure 1. Particle density and water absorption were specified according to EN 1097-3 [36], while crushing resistance was determined according to EN 13055-1 [37].

Pre-coating LWAs fractions with different cement pastes was carried out 28 days before concrete preparation. The detailed procedure for preparation of LWAs impregnated with cement paste is described in [32]. Different lightweight aggregates were submerged in cement paste for 30 min. Then, the aggregates were separated from cement slurry on sieves. The aggregates were dried out prior to impregnation. Most of them were used in oven-dry condition. For comparison reasons, to check the influence of initial LWA moisture content on effectiveness of impregnation, both Lytag fractions were also applied in a pre-moistened condition for immersion in cement pastes. The cement used for aggregate impregnation was CEM I 42.5 R. Two cement pastes *a* and *b* of similar rheology and different water-cement ratio (*w*/*c* = 0.55 and 0.37) were applied to pre-coat LWAs particles. 

**Figure 2:**
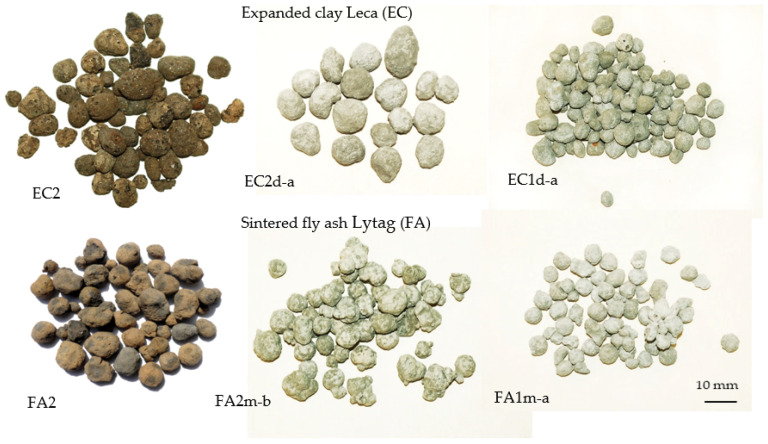
Examples of plain and impregnated lightweight aggregates used for research (LWAs symbols acc. to Table 1).

To maintain the same consistence, cement paste *b* with *w*/*c* = 0.37 was modified with superplasticizer (Sica Viscocrete). The superplasticizer (Sp) was used in the amount of 1% in relation to cement mass. The thickness of the pre-coated layer on the aggregate shell seemed to be independent on the cement paste type used for impregnation and ranged from 0.1 to 0.3 mm. The examples of various plain and impregnated aggregates used for making concretes are presented in Figure 2.

Sixteen concrete mixtures were made of sixteen different lightweight aggregates, plain and pre-coated. The other constituent materials for mix preparation were as follows: fine aggregate river normal-weight sand 0/2 mm (Figure 1), cement CEM I 42.5 R and tap water.

No admixture application was assumed. All mixes were characterized by the same volume compositions: ca. 44% of LWA (plain or impregnated) and ca. 56% of cement matrix. To maintain the comparable proportion between volume of mortar and LWA, the plain lightweight aggregates were initially pre-moistened prior to being used for concrete, while impregnated fractions were used in a dry condition. The initial moisture content of non-impregnated aggregates corresponded to their water absorption after 1 h. It was 17% and 18% for Lytag fractions, respectively, for 6/12 mm and 4/8 mm. In the case of the more porous Leca, the initial moisture content was assumed as 27% and 34%, respectively, for 8/16 mm and 4/8 mm. The compositions of all prepared concretes are given in Table 2.

Since lightweight aggregates used for concretes differed in grading and particle shapes, the consistence of fresh mixtures, tested and classified according to EN 12350-5:2019 [38] and EN 206 [11], respectively, ranged from F3–F4. 

**Figure 3:**
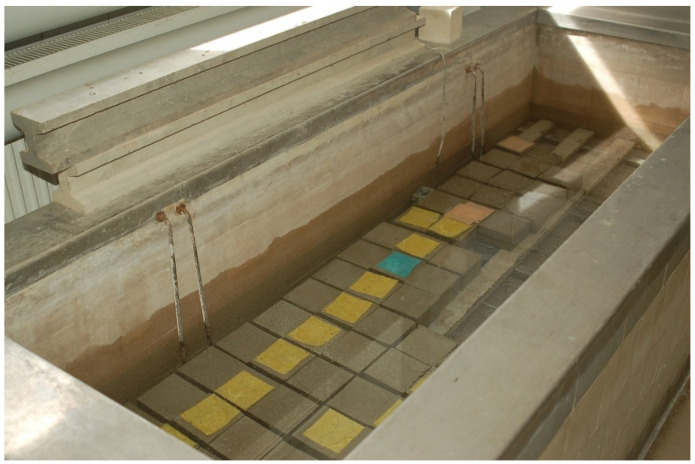
Curing cube specimens in water until testing.

Nine cube specimens, with side of 100 mm, were cast for each composite series. The specimens were demolded after 24 h and cured in water (Figure 3) at a temperature of 20 °C, according to EN 12390-2 [39], for the next 27 days. Six specimens of each series were subject to a compressive strength test, according to EN 12390-3 [40]. The other three specimens were destined for continuous density measurements, according to EN 12390-7 [41]. The specimens previously saturated in water were dried out in an oven at a temperature of 50 °C. The specimens’ mass was weighed until two subsequent results did not differ by more than 0.2% by mass. Finally, the density measurements lasted 22 days. Such a testing procedure, besides the data on density in saturated and oven-dry density, gave information on the rate of concrete drying, which is a very important parameter for lightweight concretes used as a structural material on site. It is believed that it is the slower drying rate of lightweight concrete structures that is responsible for generating additional tensile stresses in the concrete. As a result, in the standard dedicated to designing concrete structures, EN 1992-1-1 [42], it was decided to lower the tensile strength value of lightweight concretes in relation to ordinary concretes of the same strength class. Moreover, the drying rate of lightweight concrete may be a crucial parameter of its freeze-thaw resistance, as was proved in [9]. Additionally, data delivered in density measurements were used to assess the water absorption of lightweight concretes. Due to the lack of a suitable European Standard, it was calculated according to Polish Standard PN-88/B-06250 [43], as the water contained in a specimen in saturated condition related to a specimen’s mass in an oven-dry condition, expressed as a percentage. Since water absorption is the basic parameter determining material permeability, it may directly describe the general durability of the material.

## 3. Results

### 3.1. Results of Density Measurements

**Figure 4:**
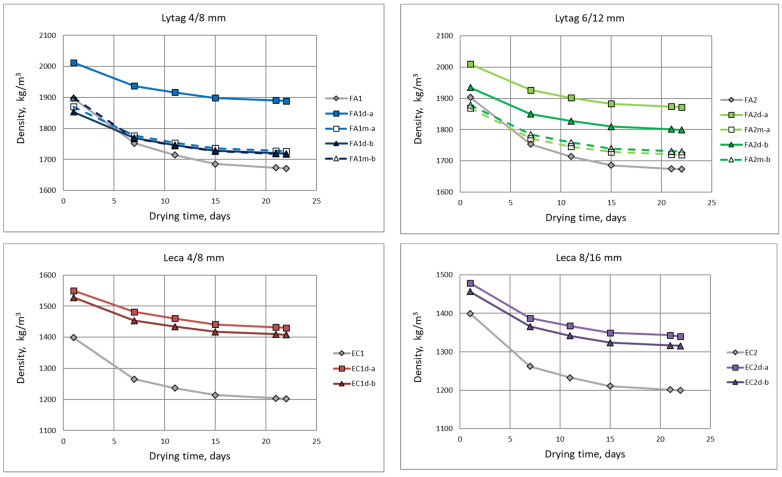
Changes in concrete density over time during drying.

**Figure 5:**
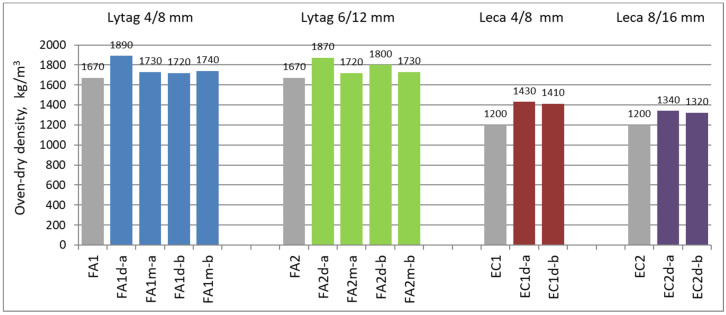
Mean oven-dry density of concretes with plain and impregnated lightweight aggregates.

The density measurements were carried out on three specimens for each concrete series. The mean density of concretes tested in saturated condition ranged from 1400 to 2010 kg/m^3^, while for oven-dry conditions the corresponding density values were 1200 to 1890 kg/m^3^. The lowest values from these ranges were obtained by reference concretes made of plain Leca aggregates, whereas the highest were reached by composites prepared with impregnated Lytag aggregates. Changes of mean density during the drying process are presented in Figure 4. The final densities in the oven-dry conditions are shown in Figure 5. The charts in Figure 4 reflects both the difference in moisture content of various concretes and the rate of their drying. Generally, all concretes made of non-impregnated aggregates revealed more dynamic moisture content changes and their later stabilization. 

The spread of individual results of density determined in any condition should be assessed as low. Coefficient of variation, defined as a ratio of standard deviation to an average value, in the case of density measurements ranged from 0 to 0.02. Individual density measurement differed from a mean value by no more than 30 kg/m^3^.

### 3.2. Water Absorption Results

**Figure 6:**
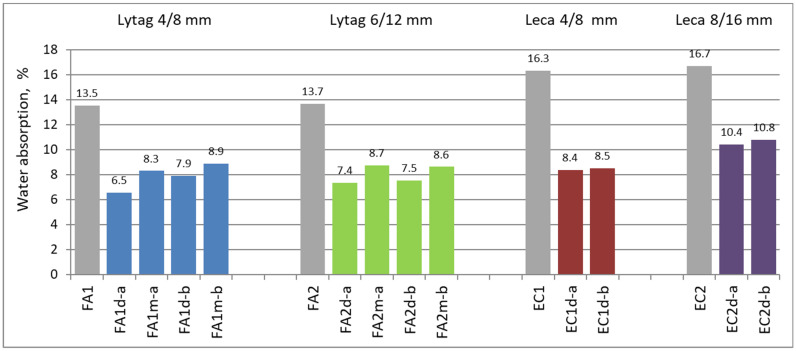
Mean water absorption of concretes with plain and impregnated lightweight aggregates.

Water absorption tests were carried out on three specimens for each concrete series. The mean values of the absorption for tested composites ranged from 6.5% for LWAC with pre-coated Lytag up to 16.7% for reference concretes with non-impregnated Leca. In general, all concretes with impregnated lightweight aggregates showed significantly lower water absorption in comparison to reference composites. The average results for water absorption measurements for all tested concretes are given in Figure 6. 

Individual measurements of water absorption differed from the mean value by no more than 0.1%. As a result, the coefficient of variation for water absorption tests varied in a very narrow range of 0 to 0.01.

### 3.3. Compressive Strength Results

**Figure 7:**
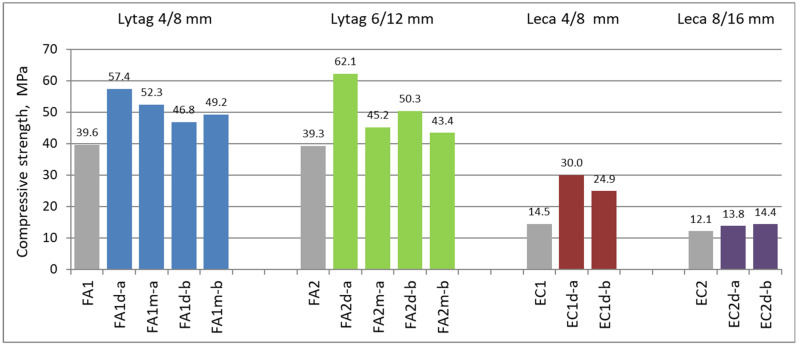
Mean compressive strength of concretes with plain and impregnated lightweight aggregates.

The compressive strength tests were specified on six specimens for each concrete series. The average strength values for tested composites ranged from 12.1 MPa for the reference concrete made of plain Leca aggregate to 62.1 MPa for the concrete made of pre-coated Lytag aggregate. Generally, composites prepared with LWAs pre-coated with cement pastes showed higher compressive strength in comparison to reference LWACs with plain aggregates. Mean compressive strength results are shown in Figure 7.

The spread of individual measurements of compressive strength may be assessed as low to moderate. Coefficient of variation for compressive strength varied from 0.01 to 0.06. The higher values from this range were revealed by concretes prepared with expanded clay aggregate, especially those with 8/16 mm. It should be stated that all composites prepared with pre-coated LWAs showed a results range smaller than reference composites with plain aggregate.

## 4. Discussion

### 4.1. Density and Its Changes during Drying

In the case of lightweight concretes, the analysis of density and its changes due to the drying process are two of the basic issues that should be taken into consideration when designing LWAC structures.

**Figure 8:**
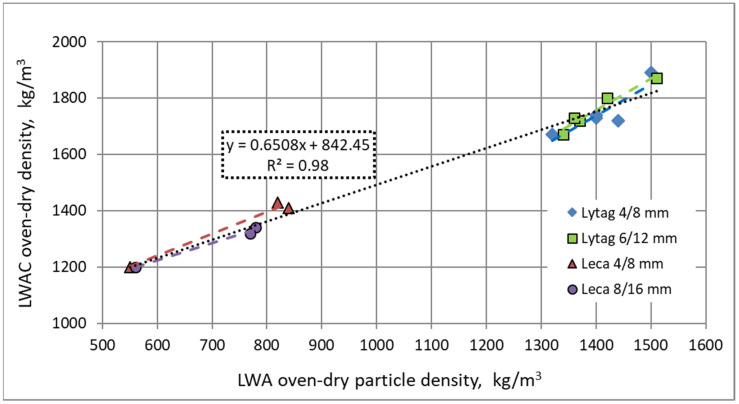
Relationship between mean oven-dry particle density of aggregates and mean oven-dry density of composites.

Due to higher particle density of aggregates pre-coated with cement pastes, all composites made of impregnated LWAs showed increased density in comparison to reference concretes prepared with plain aggregates. As a result, there is a clear relationship between aggregate oven-dry particle density and the final oven-dry density of a composite, regardless of whether impregnated or plain aggregate was used (Figure 8). High linear correlation between LWA and LWAC densities is proof that it was the aggregate type only that differed the compositions of particular composites. 

The increase in oven-dry density resulting from the application of pre-coated aggregates instead of plain LWAs ranged from 3% to 19%. The smallest increase (3–4%) was revealed for concretes made of aggregates pre-coated in moist condition. When aggregates were pre-coated as initially dry, the density increase varied from 8% to 13% for all concretes, except those made of Leca 4/8 mm, which showed higher densities by 18–19% in comparison to reference composites. This bigger increase in density is caused by the specific open-pore structure of this fraction, and its ability to absorb high cement paste content during impregnation. Therefore, it corresponds to the highest increase in particle density for Leca 4/8 mm subject to impregnation (Table 1). The cement paste type used for aggregate impregnation also had a certain impact on concrete density. Despite the consistency of both cement pastes being similar, aggregates pre-coated with cement paste *a* (*w*/*c* = 0.55) showed higher density in comparison to those impregnated with the denser cement paste *b* (*w*/*c* = 0.37). As a result, concretes made with aggregates pre-coated with cement paste *a* also showed a slightly bigger increase in density in comparison to those made of LWAs pre-coated with slurry *b*. The probable explanation of this phenomenon is a special mechanism of cement paste absorption by the porous aggregate. As it was stated in [32,44,45], the aggregate is likely to absorb water from cement paste first, and then the water-filled LWA pores are mixed with cement slurry on the basis of diffusion. In such a situation, the lower content of water in cement paste *b* could not give such effective impregnation as in the case of the application of the weaker and lighter cement paste *a*.

Due to the much higher initial water absorption of applied aggregates, the above increase in density for tested composites turned out to be greater than what was achieved for concretes with impregnated LWAs, as discussed in [13], or concretes prepared with pre-coated RCAs, as described in [15,31]. However, when considering aggregates of similar structure, even if they were impregnated with polymers [17], the achieved results of concrete density seem to be comparable or even lower.

As it was shown in Figure 4, the dynamic of lightweight composites drying differed depending mainly on the type of aggregate used, and whether the aggregate was impregnated or not. In the case of concretes with pre-coated aggregates, LWA initial moisture content before impregnation and the type of cement paste used for impregnation also turned out to be important. Generally, pre-coating of lightweight aggregates protected concretes from a high moisture content and led to quicker drying. This fact is especially important considering the negative consequences of the long drying process of lightweight concrete in construction elements. Taking into consideration that in the case of tested concretes the drying process in temperature of 50 °C for relatively slim elements (100 mm) lasted over twenty days, it should be stated that in practice the drying time for LWAC structures may be even longer due to typically lower curing temperatures and thicker elements. To sum up, application of pre-coated aggregates may reduce the risk of freeze-thaw destruction, when concrete matures in a winter condition, and may limit the risk of generation of additional tensile stresses resulted from uneven drying. 

The observed density increment of concretes prepared with aggregates pre-coated with cement paste was greater than indicated in [13] for concretes with impregnated LWAs, or in [15,31] for concretes made of impregnated RCAs due to significantly lower initial water absorption of those aggregates. However, the achieved increase in density was similar or lower when compared to concretes with aggregates of higher initial water absorption, even when they were pre-coated with polymers [17].

### 4.2. Water Absorption 

The water absorption is one of the most basic indicators of general concrete durability as it is directly connected with composite permeability. Although the results of water absorption achieved for tested concretes, especially those made of plain aggregates, were bigger in comparison to typical normal-weight composites, all LWACs fulfilled the criterion determined in [46], which requires that the water absorption for unprotected concrete exposed to direct weather conditions be less than 20%. Nevertheless, it is well proved [9] that lightweight concretes with higher water absorption reveals lower freeze-thaw resistance, greater carbonation depth and chloride ions penetration.

As it can be seen in Figure 6, pre-coating lightweight aggregates with cement pastes caused a significant reduction in water absorption for all concretes regardless of the types of LWA and cement paste used for impregnation, as well as of initial aggregate moisture content before pre-coating.

The decrease in water absorption for concrete made of pre-coated aggregates ranged from 34% to 52% in relation to reference composites prepared with plain aggregates. Pre-coating the aggregates of smaller fraction (4/8 mm) of both types of aggregates seems to be the most effective for the improvement of composite water tightness. In this case, the share of LWA external shell sealed with cement paste in whole particle volume was much greater than for the bigger fraction. As a result, concretes with the largest pre-coated aggregate (Leca 8/16 mm) showed a relatively lower decrease in water absorption. The initial LWAs moisture content and the cement type used for aggregate impregnation also had an impact on the decrease in water absorption. The lower initial moisture content of aggregate prior to pre-coating led to tighter aggregate shell and, as a consequence, to tighter composite structure. Application of cement paste *a* of higher *w*/*c* (0.55) for aggregate pre-coating resulted in slightly lower water absorption than in the case of slurry *b* of *w*/*c* = 0.37. This observation is consistent with that made at density analysis (see Section 4.1). 

**Figure 9:**
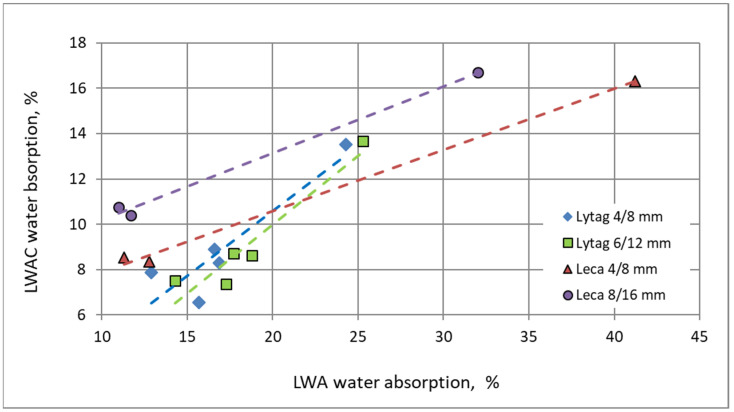
Relationship between mean water absorption of aggregates and water absorption of composites.

In opposition to density, there is no clear general relationship between water absorption of aggregate and water absorption of the final composite (Figure 9). Pre-coating aggregates with cement paste gave reduction in water absorption for both LWAs and LWACs. Nevertheless, the reduction tendencies differed not only quantitatively but also qualitatively. In comparison to Lytag, impregnation of Leca aggregates was much more effective for improvement of aggregate water tightness (Table 1). Meanwhile, there are not so many considerable differences in the water absorption reduction of composites. The most important factor influencing the water absorption of the composite seems not to so much be the water absorption of the aggregate but whether the aggregate was impregnated or not. 

**Figure 10:**
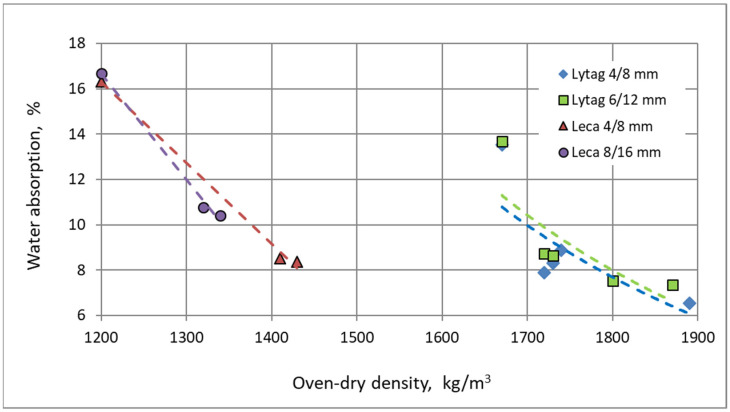
Relationship between mean oven-dry density of tested concretes and their mean water absorption.

Although the concrete oven-dry density is an indicator of porosity of the composite, the general correlation between oven-dry density and water absorption for all tested LWACs is very weak (Figure 10). The influence of aggregate pore structure, especially the tightness of its external shell, strongly disturbs this relationship.

The comparison of effectiveness of aggregate impregnation applied in this study and other research [13,28,31] on the limitation of water absorption is not reliable due to different testing procedures and various types of aggregates. It seems that pre-coating with cement paste may be at least as effective for composite sealing as in the case of polymers application. Nevertheless, to prove this thesis it is necessary to carry out some comparative tests.

### 4.3. Compressive Strength 

Compressive strength, as the most important mechanical property of concrete, shows its suitability for structural applications. Moreover, together with LWAC density, it is the base for the estimation of other mechanical properties of lightweight composites. 

The compressive strength of reference composites indicates that used Lytag aggregate is more appropriate for structural concretes than applied Leca (Figure 7). To obtain structural concrete with this type of expanded clay, it would be necessary to use cement matrix of much higher strength and/or in bigger proportion to aggregate than was applied in this research.

Pre-coating lightweight aggregates with cement pastes significantly influenced compressive strength of composites. The strength increase ranged from 11% up to 107%. The most pronounced strength increase was observed in the case of concrete with Leca 4/8 mm impregnated with cement paste *a* (*w*/*c* = 0.55), due to its deepest penetration into aggregate particles and their effective strengthening. Impregnation of dry Lytag aggregates led to compressive strength increase by 18–58%. Application of cement paste *a* for Lytag aggregate impregnation also turned out to strengthen the particles the most. Initially moistening aggregate particles prior to their pre-coating resulted in concrete strength visibly higher, by up to 32%, than for reference composites made of plain aggregates, on the one hand. On the other hand, the strength of concretes made of initially moistened pre-coated LWAs were mostly considerably lower, even by 28%, when compared to composites with initially dry aggregates. 

The separate analysis is required for composites with Leca 8/16 mm. Since this fraction is characterized by the biggest size and a very high porosity (80%), tightening external shell of LWAs particles in an impregnation process, although it considerably enhanced composite water absorption (see Section 4.2), nevertheless, it could not influence the strength so significantly as it took place in the case of the application of the other lightweight aggregates of a smaller size. 

**Figure 11:**
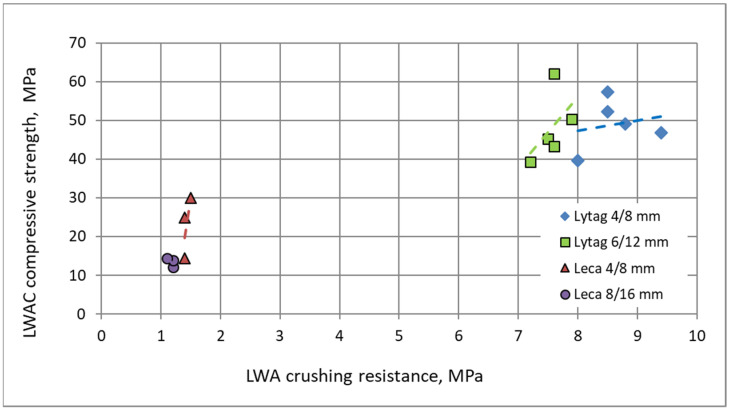
Relationship between mean crushing resistance of lightweight aggregates and mean compressive strength of composites.

Analyzing the relationship between mean LWAs crushing resistance and mean LWACs compressive strength (Figure 11), it should be stated that there is no general correlation for these properties within the confines of all concrete series. However, a certain tendency may be observed for individual types of aggregate. Except Leca 8/16 mm, even a minor enhancement in crushing resistance of lightweight aggregate, resulted from impregnation, corresponded in incomparable higher compressive strength of composites. Therefore, crushing resistance of pre-coated aggregate cannot be treated as a reliable indicator of composite strength. 

**Figure 12:**
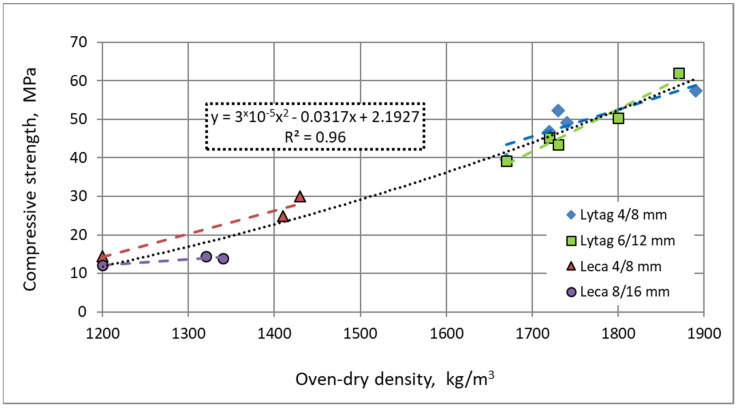
Relationship between mean oven-dry density of tested concretes and their mean compressive strength.

In contrast to water absorption, there is a direct general relationship between compressive strength of lightweight composites and their oven-dry density (Figure 12). In this case, the influence of the used aggregate type is of less importance due to the fact that the general concrete tightness (defined as the ratio of apparent and specific density) seems to be the dominant factor affecting strength.

**Figure 13:**
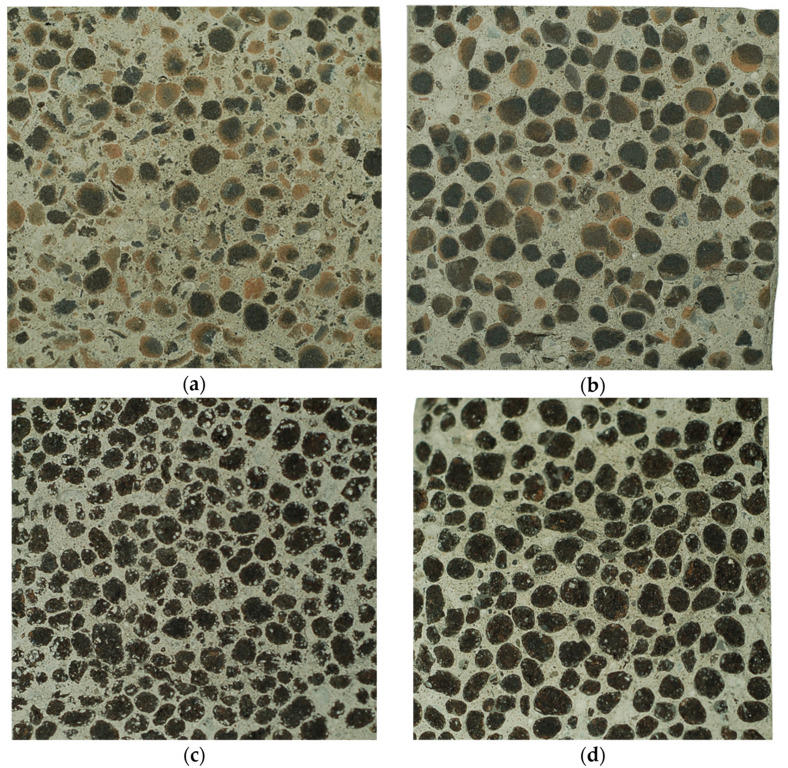
Examples of fracture in composite cube specimens with sides of 100 mm subject to splitting, (**a**) non-impregnated sintered fly ash (FA1), (**b**) impregnated sintered fly ash (FA1d-b), (**c**) non-impregnated expanded clay (EC1), (**d**) impregnated expanded clay (EC1d-a).

When excluded results for composites with Leca 8/16 mm, the strength values achieved in this research indicate that impregnation of applied LWAs with cement pastes was much more effective in composite strength improvement than it was reported in the research described in [13,15,17,19,24,31,33]. This high effectiveness resulted from a relatively open structure of used aggregates, higher strength of cement paste compared to other impregnant substances applied for aggregate pre-coating and perfect material compatibility between cement paste cover of impregnated LWAs and cement matrix in concrete. The proof for a good bond between pre-coated aggregates and cement matrix in concrete is the way of fracturing in composite under loading. In the case of all tested concretes made of impregnated aggregates, regardless of the aggregate and cement paste types as well as LWAs initial moisture content prior to pre-coating, at failure the cracking occurred through the aggregate grains instead of the contact zone between the aggregate and the cement matrix (Figure 13). 

**Figure 14:**
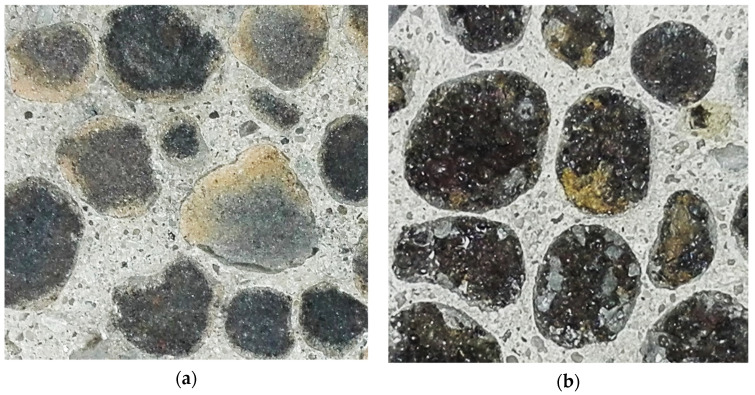
Bond between cement matrix and impregnated lightweight aggregates (4/8 mm) in concretes at fracture under compressive load: (**a**) sintered fly ash and (**b**) expanded clay.

Meanwhile, in the case of reference concretes made of pre-moistened non-impregnated Lytag it happened that individual LWA particles detached from the cement matrix. As a result, in Figure 13, the number of LWA grains visible in the fracture of concrete with non-impregnated sintered fly ash aggregate (FA1) is lower than in the case of a composite with this same aggregate but pre-coated (FA1d-b). For concretes with Leca aggregates, due to their more porous external shell, such differences did not appear. In spite of the good adhesion of cement paste to all pre-coated aggregates, the appearance of the bond in Lytag and Leca composites is different (Figure 14). The impregnated cover on sintered fly ash particles is difficult to distinguish, while in the case of expanded clay, big pores filled with cement paste used for pre-coating are clearly visible. The analysis under optical microscope showed that the penetration of cement paste into LWAs pores took place to the depth up to 0.5 and 3.0 mm, respectively, for Lytag and Leca aggregates.

## 5. Conclusions

The research program carried out and the analysis of tests results proved high effectiveness of impregnation of lightweight aggregates with cement pastes in the improvement of composite properties related to both durability and mechanical characteristics. In particular, the following main conclusions can be drawn from this research:

- The most crucial factors determining the effectiveness of impregnation of lightweight aggregates with cement pastes in modifying the properties of concrete composites were the aggregate type (its porosity structure) and its size. Other parameters, such as the composition of impregnating slurry and the initial moisture content of LWA before pre-coating, also mattered.
- Selecting pre-coated aggregates with cement paste resulted in relatively slight increase in concrete density (by up to 19%), on the one hand. On the other hand, it caused a very significant reduction (by up to 52%) in composite water absorption and an incomparably greater (by up to 107%) growth in compressive strength. Due to an overly large size and the high porosity of particles of Leca 8/16 mm, concretes with this pre-coated aggregates turned out to be the only exceptions, where impregnation did not affect such a pronounced increase in strength (only up to 19%).
- Contrary to expectations, the lower water-cement ratio of the slurry used for pre-coating lightweight aggregates was not conducive to increasing the efficiency of LWA impregnation in improving the properties of concrete composites. The probable explanation of this phenomenon is a special mechanism of cement paste absorption by the porous aggregate.
- Application of lightweight aggregates in moistened condition for pre-coating treatment significantly reduced the effectiveness of impregnation in the modification of the properties of LWACs. The water absorption decrease was reduced up to 39%, while the strength increase was limited to 32%.
- Due to the specificity of tests, except density, there is no direct quantitative relationship between the properties of LWAs pre-coated with cement paste and the properties of concrete composites made of these aggregates.

Summing up, pre-coating lightweight aggregates with cement paste seems to be a very effective method to limit water content in fresh, as well as young, LWAC and to enhance both the durability and mechanical characteristics of concrete composites. Compared to aggregate pre-soaking, such an initial treatment of LWAs may lead to wider possibilities to apply lightweight aggregate composites as a structural concrete characterized by improved performance during its lifetime. Nevertheless, since the impregnation procedure results in a higher cost of each concrete unit in relation to a composite prepared with plain LWA, it should be taken into consideration mainly in the following circumstances: the aggregate is characterized by external shell with relatively open pore structure; the size of aggregate is limited when its porosity is comparatively high; and other types of lightweight aggregate, which could be able to provide concrete of specified properties, are not available. 

Further research should be focused on the application of mineral additives in cement slurries to be used for LWAs impregnation in order to reduce the total cement content in concrete. Some direct comparative tests on LWACs with aggregates pre-coated with cement paste and other popular impregnants should also be carried out.

## Figures and Tables

**Table 1:** Properties of plain and pre-coated lightweight aggregates used in the research.

LWA Designation	LWA Type	Fraction	Cement Paste Used for LWA Impregnation	Initial Moisture Content before Impregnation, %	Particle Density, kg/m^3^	Water Absorption after 24 h, %	Max. Water Absorption, %	Crushing Resistance, MPa
FA1	Lytag	4/8 mm	-	-	1320	18.8	24.3	8.0
FA1d-a	Lytag	4/8 mm	*w*/*c* = 0.55; Sp = 0	0	1500	12.4	15.7	8.5
FA1m-a	Lytag	4/8 mm	*w*/*c* = 0.55; Sp = 0	18.0	1400	13.7	16.9	8.5
FA1d-b	Lytag	4/8 mm	*w*/*c* = 0.37; Sp = 1%m.c.	0	1440	9.8	12.9	9.4
FA1m-b	Lytag	4/8 mm	*w*/*c* = 0.37; Sp = 1%m.c.	18.0	1400	12.8	16.6	8.8
FA2	Lytag	6/12 mm	-	-	1340	19.3	25.3	7.2
FA2d-a	Lytag	6/12 mm	*w*/*c* = 0.55; Sp = 0	0	1510	14.2	17.3	7.6
FA2m-a	Lytag	6/12 mm	*w*/*c* = 0.55; Sp = 0	17.0	1370	14.9	17.7	7.5
FA2d-b	Lytag	6/12 mm	*w*/*c* = 0.37; Sp = 1%m.c.	0	1420	11.7	14.3	7.9
FA2m-b	Lytag	6/12 mm	*w*/*c* = 0.37; Sp = 1%m.c.	17.0	1360	14.2	18.8	7.6
EC1	Leca	4/8 mm	-	-	550	36.4	41.2	1.4
EC1d-a	Leca	4/8 mm	*w*/*c* = 0.55; Sp = 0	0	820	11.7	12.8	1.5
EC1d-b	Leca	4/8 mm	*w*/*c* = 0.37; Sp = 1%m.c.	0	840	10.2	11.3	1.4
EC2	Leca	8/16 mm	-	-	560	30.7	32.0	1.2
EC2d-a	Leca	8/16 mm	*w*/*c* = 0.55; Sp = 0	0	780	10.8	11.7	1.2
EC2d-b	Leca	8/16 mm	*w*/*c* = 0.37; Sp = 1%m.c.	0	770	10.2	11.0	1.1

**Table 2:** Compositions of lightweight concretes prepared with plain and pre-coated LWAs.

N°	Mix Designation	LWA, kg/m^3^	Water to LWA, kg/m^3^	Natural Sand, kg/m^3^	Cement, kg/m^3^	Water, kg/m^3^
1	FA1	581	99	512	420	231
2	FA1d-a	660	-	512	420	231
3	FA1m-a	616	-	512	420	231
4	FA1d-b	634	-	512	420	231
5	FA1m-b	616	-	512	420	231
6	FA2	590	100	512	420	231
7	FA2d-a	664	-	512	420	231
8	FA2m-a	603	-	512	420	231
9	FA2d-b	625	-	512	420	231
10	FA2m-b	598	-	512	420	231
11	EC1	242	82	512	420	231
12	EC1d-a	361	-	512	420	231
13	EC1d-b	370	-	512	420	231
14	EC2	246	66	512	420	231
15	EC2d-a	343	-	512	420	231
16	EC2d-b	339	-	512	420	231

## Data Availability

Not applicable.
